# Supplementary material for: Heterogeneous response of endothelial cells to insulin-like growth factor 1 treatment is explained by spatially clustered sub-populations
Source: Biol Open. 2019 Nov 4;8(11):bio045906. doi: 10.1242/bio.045906 (PMC6899026; doi:10.1242/bio.045906)
Supplement: Supplementary information [file biolopen-8-045906-s1.pdf]

Biology Open (2019): doi:10.1242/bio.045906: Supplementary information

SUPPLEMENTARY INFORMATION

Heterogeneous response of endothelial cells to insulin like growth factor 1 treatment is explained by spatially clustered subpopulations

Christina Kim[a,b,d], Gregory J Seedorf[b,c], Steven H Abman[b,c], Douglas P Shepherd[b,d,e,*]

a. Department of Surgery, University of Colorado Anschutz Medical Campus, Aurora, CO 80045

b. Pediatric Heart Lung Center, University of Colorado Anschutz Medical Campus, Aurora, CO 80045

c. Department of Pediatrics, University of Colorado Anschutz Medical Campus, Aurora, CO 80045

d. Department of Pharmacology, University of Colorado Anschutz Medical Campus, Aurora, CO 80045

e. Department of Physics and Center for Biological Physics, Arizona State University, Tempe, AZ 85287

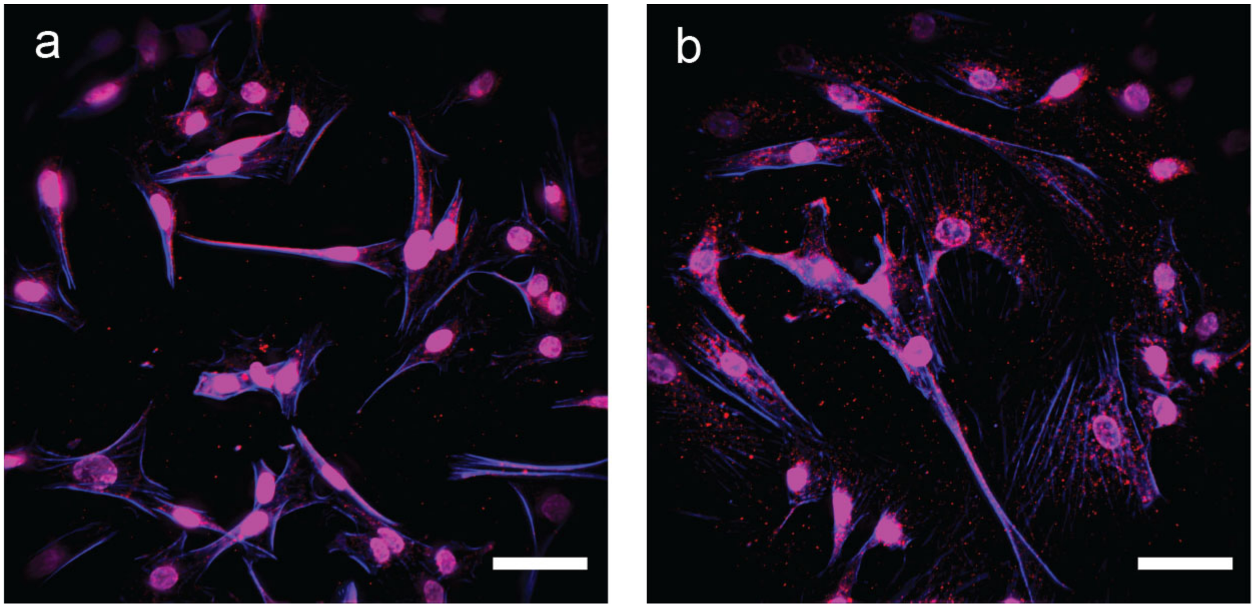

Figure S1. vWF expression for a) normal and b) PPHN PAEC. (scale bar - 50 um)

Biology Open • Supplementary information

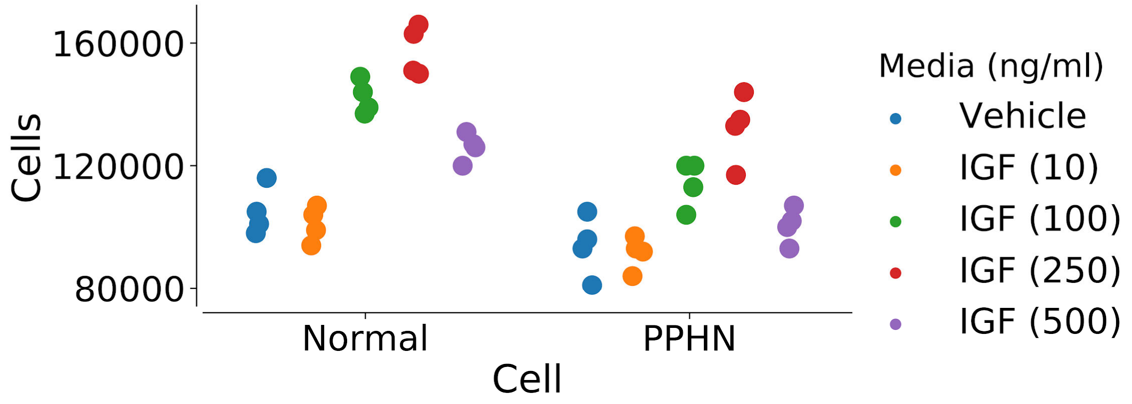

Figure S2. IGF-1 dose response for normal and PPHN PAEC (one day of growth).

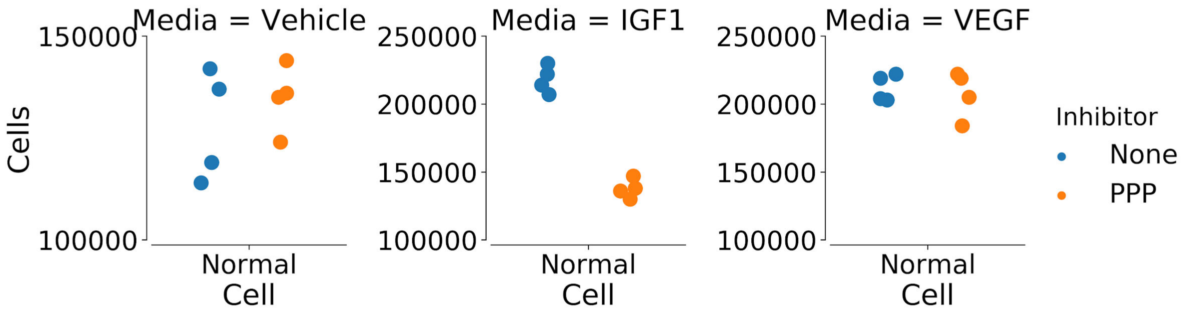

Figure S3. Normal PAEC growth in the presence of 25 nM picropodophyllin (PPP) in vehicle, IGF-1 (250 ng/ml), and VEGF (50 ng/ml) media (one day of growth).

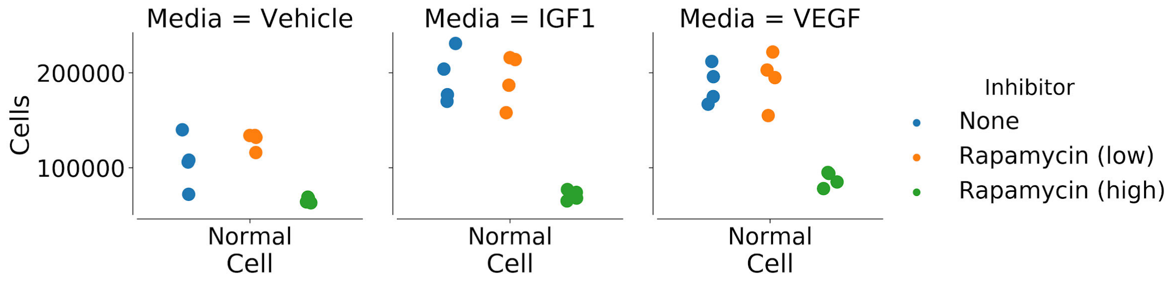

Figure S4. Normal PAEC growth in the presence of low (.01 nM) and high (.1 nM) rapamycin (RP) in vehicle, IGF-1 (250 ng/ml), and VEGF (50 ng/ml) media (one day of growth).

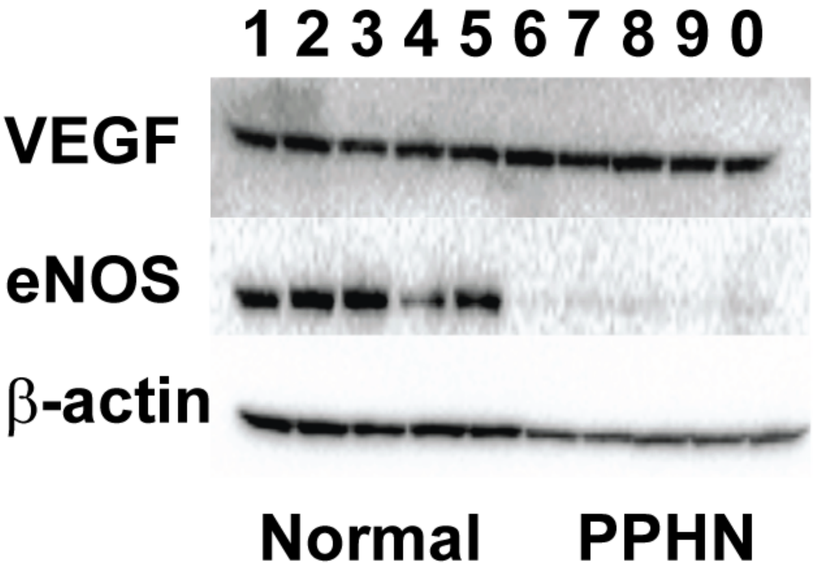

Figure S5. One of three western blot experiments for data in Figure 3b. Key: (1 – 0 hour, normal PAEC; 2 – 1 hour, normal PAEC, vehicle media; 3 – 1 hour, normal PAEC, IGF-1 media; 4 – 24 hour, normal PAEC, vehicle media; 5 – 24 hour, normal PAEC, IGF-1 media; 6 – 0 hour, PPHN PAEC; 7 – 1 hour, PPHN PAEC, vehicle media; 8 – 1 hour, PPHN PAEC, IGF-1 media; 9 – 24 hour, PPHN PAEC, vehicle media; 0 – 24 hour, PPHN PAEC, IGF-1 media. There were no significant deviations observed in the two replicates of this experiment.
